# Phylogenetic analysis and phenotypic characterisatics of two Tibet EV-C96 strains

**DOI:** 10.1186/s12985-019-1151-7

**Published:** 2019-03-29

**Authors:** Lan Hu, Yong Zhang, Mei Hong, Qin Fan, Dongmei Yan, Shuangli Zhu, Dongyan Wang, Wenbo Xu

**Affiliations:** 1 WHO WPRO Regional Polio Reference Laboratory and NHC Key Laboratory of Biosafety, National Institute for Viral Disease Control and Prevention, Chinese Center for Disease Control and Prevention, Beijing, People’s Republic of China; 2 Department of the Laboratory, Guanghua Hospital of Traditional and Western Medicine, Changning District, Shanghai, People’s Republic of China; 3 Tibet Center for Disease Control and Prevention, Lhasa City, Tibet Autonomous Region People’s Republic of China; 4 Zhejiang Center for Disease Control and Prevention, Hangzhou city, Zhejiang Province People’s Republic of China; 5 Anhui University of Science and Technology, Hefei city, Anhui Province People’s Republic of China

**Keywords:** Enterovirus C96, Cell sensitivity, Phylogenetic analysis, Recombinant

## Abstract

**Background:**

Enterovirus C96 (EV-C96) is a newly named type of enterovirus belonging to species C, and the prototype strain (BAN00–10488) was firstly isolated in 2000 from a stool specimen of a patient with acute flaccid paralysis in Bangladesh. In this study, we report the genomic and phenotypic characteristics of two EV-C96 strains isolated from individuals from the Tibet Autonomous Region of China.

**Methods:**

Human rhabdomyosarcoma (RD), human laryngeal epidermoid carcinoma (HEp-2), and human cervical cancer (Hela) cells were infected with the Tibet EV-C96 strains, and enterovirus RNA in the cell culture was detected with a real time RT-PCR-based enterovirus screening method. The temperature sensitivity of Tibet EV-C96 strains were assayed on a monolayer of RD cells in 24-well plates. Full-length genome sequencing was performed by a ‘primer-walking’ strategy, and the evolutionary history of EV-C96 was studied by maximum likelihood analysis.

**Results:**

Strain 2005-T49 grew in all three kinds of cells, and it was not temperature sensitive. In contrast, none of the three cells produced CPE for strain 2012-94H. Phylogenetic analysis of the two Tibetan viruses, other EV-C96 strains, and EV-C prototypes showed that EV-C96 strains were grouped into three clusters (Cluster1–3) based on their *VP1* sequences, which may represent three genotypes. Phylogenetic trees based on the *P2* and *P3* regions highlighted the difference between Chinese EV-C96 strains and the EV-C96 prototype strain BAN-10488. All Chinese strains formed a cluster separate from BAN-10488, which clustered with CV-A1/CV-A22/CV-A19.

**Conclusions:**

There is genetic variability between EV-C96 strains which suggest that at least few genetic lineages co-exist and there has been some degree of circulation in different geographical regions for some time. Some recombination events must have occurred during EV-C96 evolution as EV-C96 isolates cluster with different EV-C prototype strains in phylogenetic trees in different genomic regions. However, recombination does not seem to have occurred frequently as EV-C96 isolates from different years and locations appear to cluster together in all genomic regions analysed. These findings expand the understanding of the characterization of EV-C96 and are meaningful for the surveillance of the virus.

## Background

Enteroviruses (EVs) are small, non-enveloped, single-stranded RNA viruses belonging to the family *Picornaviridae*, which can be classified to 12 species, including nine enteroviruses, Enterovirus A (EV-A) to EV-H, and EV-J and three rhinoviruses, Rhinovirus A–C [1]. Species EV-A–D correspond to the enteroviruses formerly named Human enterovirus A–D, and species EV-C currently consists of 23 serotypes: three polioviruses (PV) type 1–3, nine group A coxsackieviruses (CV-A1, A11, A13, A17, A19–22, and A24), and 11 new EV-C types, including EV-C95, EV-C96, EV-C99, EV-C102, EV-C104, EV-C105, EV-C109, EV-C113, and EV-C116–C118 [2–7]. EV-C has been correlated with a wide variety of clinical manifestations, ranging from mild respiratory infections to severe central nervous system infections, such as acute flaccid paralysis (AFP) and acute haemorrhagic conjunctivitis [8, 9]. Generally, enterovirus genomes contain approximately 7500 nucleotides consisting of a single open reading frame that is flanked by 5′ and 3′ untranslated regions (5′- and 3′-UTRs). The single open reading frame is translated as a single polypeptide that is then autocatalytically cleaved to yield three polyprotein precursors: P1, P2, and P3. Polyprotein P1 is further cleaved to generate capsid proteins VP1–VP4, and P2 and P3 are cleaved to generate non-structural proteins named 2A–2C and 3A–3D, respectively.

The molecular typing method used in recent years to identify different types of enteroviruses is equivalent to the formerly used neutralisation test [10, 11]. The recommended criteria for the molecular typing method are based on the genetic diversity of *VP1* region: EVs that share > 75% nucleotide identity and > 88% amino acid identity in the *VP1* region are classified as the same type [11, 12].

EV-C96 is a recently described serotype in the EV-C species based on the molecular typing method [13] and the prototype strain (BAN00–10488) was first isolated in 2000 from a stool specimen of a patient with AFP in Bangladesh [5]. Subsequently, several other EV-C96 strains were isolated from patients with AFP or healthy individuals (asymptomatic infections) in Finland, Slovakia, the Philippines, Cambodia, China, and Bolivia [13–19]. Here, we report the complete genome sequences of two EV-C96 strains, 2005-T49/XZ/CHN/2005 (hereafter referred to as 2005-T49) and 2012-94H/XZ/CHN/2012 (hereafter referred to as 2012-94H), which were isolated in Tibet, China. Currently, 41 *VP1* sequences, including 11 entire *VP1* sequences and 9 complete genome sequences, of EV-C96 strains are available in GenBank, which we used to perform an evolutionary analysis of EV-C96. This study expands the number of EV-C96 full-length genome sequences in GenBank and provides valuable information regarding the molecular epidemiology of EV-C96.

## Methods

### Sample collection

Two stool samples were collected in the Tibet Autonomous Region in 2005 and 2012, respectively. One (2005-T49) was collected from a patient with AFP in 2005 in Lazi County in the Rikaze Prefecture of Tibet during the course of poliovirus surveillance in support of the global polio eradication initiative, and the other (2012-94H) was collected from a healthy child in Zhangmu County in Rikaze Prefecture of Tibet during an enterovirus surveillance program of healthy children in 2012.

### Viral isolation and primary identification

The stool sample (about 2 g) was dissolved in 10 ml of complete phosphate-buffered saline with antibiotics, 1 g of glass beads and 1 ml chloroform, then shaked vigorously for 20 min using a mechanical shaker. Spined for 20 min at 1500 g in a refrigerated centrifuge, and finally aspirated the supernatant for further use. 0.2 ml stool supernatant was inoculated into human rhabdomyosarcoma (RD), human laryngeal epidermoid carcinoma (HEp-2) and human cervical cancer (Hela) cells (all in Hank’s maintenance medium) and incubated at 36 °C for virus propagation, then the cells were examined for the development of EV-like cytopathic effects (CPE) daily, recorded all observations of inoculated and control cultures for at least 7 days [20]. If characteristic enterovirus CPE appeard, then stored at − 20 °C for further use; If no CPE appeard after 7 days observing, performed a blind passage and continued examination for further 7 days. Enterovirus RNA was extracted from the cell culture using the QIAamp Viral RNA Mini Kit (Qiagen, Hilden, Germany), and was detected with a real time RT-PCR-based enterovirus screening method [21]. An EV-B85 strains, HYTY-ARL-AFP02F [22], was used as positive control, the RD cell culture without inoculating any samples was used as negtive control.

### Assay for temperature sensitivity

For poliovirus vaccine strains, temperature sensitivity usually correlates with attenuation, and vaccine-derived mutant strains which have lost their temperature sensitivity have been shown to be neurovirulent, so temperature sensitivity may be an acceptable method for evaluating the presence of attenuating mutations for enteroviruses [23]. The temperature sensitivity of EV-C96 strain 2005-T49 was assayed on a monolayer of RD cells (CCL-136, ATCC, Passage number is 241) in 24-well plates [24]. We could not assess the temperature sensitivity of strain 2012-94H because it did not induce CPE in RD, HEp-2 or Hela cells. Add 100 ul of RD cells from a cell suspension containing 2 × 10^5^ cells/ml to all wells in the 24-well plate, then the plates were inoculated with 50 μL undiluted virus stocks. Two different incubators were used: one incubator was adjusted to 36 °C as the optimal temperature for virus propagation, and another one was adjusted to 39.5 °C as the supraoptimal temperature for virus propagation. After adsorption at 36 °C or at 39.5 °C for 1 h, the unadsorbed virus inoculum was removed and 100 μL of maintenance medium (2% Foetal calf serum in Eagle’s minimum essential medium) was added to each well. The plates were continually incubated at 36 °C or at 39.5 °C and harvested at 7 time points post-infection (8 h, 16 h, 24 h, 48 h, 72 h, 96 h, and 120 h) in succession. The 50% cell culture infectious dose (CCID_50_) was calculated by the Spearmann-Kärber end-point dilution method on monolayer RD cells in 96-well plates at 36 °C. Two Xinjiang EV-B85 strains, HYTY-ARL-AFP02F, which is not temperature sensitive, and HT-LYKH202F, which is temperature sensitive, [22] were used as experimental controls to ensure that the cells and viral replication were not affected at high temperature under the experimental conditions. Virus isolates showing more than a two-log reduction in titre at the elevated temperature were considered to be temperature sensitive [25].

### Full-length genome sequencing

HEp-2 cell cultures of 2005-T49 (CPE observed) and 2012-94H (real-time RT-PCR positive although no CPE observed) were frozen three times to release viruses. For molecular typing, viral RNA was extracted from 140ul HEp-2 cell culture using the QIAamp Viral RNA Mini Kit (Qiagen, Hilden, Germany) and stored at − 80 °C until use. RT-PCR was performed to amplify the *VP1*-coding region using the PrimeScript™ One Step RT-PCR Kit Ver.2 (TaKaRa, Dalian, China) with primers E292 and E222 [26]. The RT-PCR products were purified using the QIAquick PCR Purification Kit (Qiagen, Hilden, Germany), and then used for nucleotide sequencing. The EV serotype was determined according to a previously described molecular typing method [12]. The primers used for amplification and sequencing were designed by a ‘primer-walking’ strategy (Table 1). Viral RNA extraction, RT-PCR amplification, and PCR product purification were performed as described above. Sequencing was performed in both directions using an ABIPRISM 3130 Genetic Analyzer (Applied Biosystems), and every nucleotide position was sequenced at least twice in order to ensure the accuracy of sequences.

**Table 1:** PCR and sequencing primers

Primer	Position	Primer sequence (5′-3′)	Direction	Reference
0001S48		GGGGACAAGTTTGTACAAAAAAGCAGGCTTTAAAACAGCTCTGGGGTT	Forward	[43]
EV-C96-1075A	1056–1075	GCCACTCTCCATAGGCAACT	Reverse	This study
EV-C96-618S	618–638	TCATAAAGCGAATTGGATTGG	Forward	This study
EV-C96-1575A	1555–1575	GGCACTATTGTTGGTTCTCAG	Reverse	This study
EV-C96-1186S	1186–1206	AAAGGTTGGTGGTGGAAATTA	Forward	This study
EV-C96-2318A	2298–2318	ACACATCTGCGGTACACACT	Reverse	This study
EV-C96-2137S	2137–2156	TGTGGTAGTATGATGGCCAC	Forward	This study
EV-C96-3331A	3312–3331	CTTGGACACCACACCCTGAT	Reverse	This study
EV-C96-3087S	3087–3107	CCACCGAGAATGTCTGTACCA	Forward	This study
EV-C96-4396A	4374–4396	TGAAGAGCACCTCTTGTTGTTC	Reverse	This study
EV-C96-4148S	4148–4168	TTACGTCATGAGACAGGGTGA	Forward	This study
EV-C96-5337A	5318–5337	AAATTGTCATCGCCCTGTTC	Reverse	This study
EV-C96-5121S	5121–5140	ATAGGCAATTGCATGGAAGC	Forward	This study
EV-C96-6155A	6136–6155	GAGGACTGCTGGTTCCTTGA	Reverse	This study
EV-C96-5870S	5870–5890	ACTGCTCGCACGCTAATGTA	Forward	This study
EV-C96-6746A	6722–6746	TGCATCATACCCTGTGTAATCA	Reverse	This study
EV-C96-6551S	6551–6570	GATCGAGGCATCAAGTCTCA	Forward	This study
EV-C96-7435A	7416–7435	CCAATTCGACTGAGGTAGGG	Reverse	This study
EV-C96-7030S	7030–7049	CCCATGAGGTTGACGCTAGT	Forward	This study
7500A		GGGGACCACTTTGTACAAGAAAGCTGGG(T)_24_	Reverse	[43]

The 5′ end sequence of the genome was obtained using a 5′ rapid amplification of cDNA ends (RACE) kit (Takara Biomedicals) according to the manufacturer’s instructions. The 3′ end sequence of the genome was obtained by amplification using an oligo-dT primer (primer 7500A) as the downstream primer listed in Table 1. The 5′ end sequence of the genome was obtained from both the viral isolates and the stool samples.

### Phylogenetic and bioinformatics analyses

The nucleotide and deduced amino acid sequences of strains 2005-T49 and 2012-94H were compared to those of the prototype EV-C strains by pairwise alignment using MEGA (version 7.0.26, [27]). The evolutionary history of EV-C96 was studied by maximum likelihood analysis. The maximum likelihood phylogenetic tree was constructed by the MEGA software and inferred by Model Finder to search the best nucleotide substitution model [28]. Regions containing alignment gaps were omitted from the analysis. The branch lengths of the dendrogram were determined from the topologies of the trees and were obtained by majority rule consensus among 1000 bootstrap replicates. Bootstrap values > 80% were considered statistically significant for grouping. Similarity plots and boot scanning analyses were performed using SimPlot (version 3.5.1; Stuart Ray, Johns Hopkins University, Baltimore, MD, USA) [29]. For the similarity plot analyses, a 200-nucleotide window was moved in 20-nucleotide steps, and boot scanning analyses were performed by using the neighbour-joining method.

### Data availability and nucleotide sequence accession numbers

The full-length genome sequences of the two Tibetan EV-C96 strains described in this study, 2005-T49/XZ/CHN/2005 and 2012-94H/XZ/CHN/2012, were deposited in the GenBank under the respective accession numbers KP984753 and KP984754.

## Results

### The two Tibetan EV-C96 strains showed different growth ability in cells

**Fig. 1:**
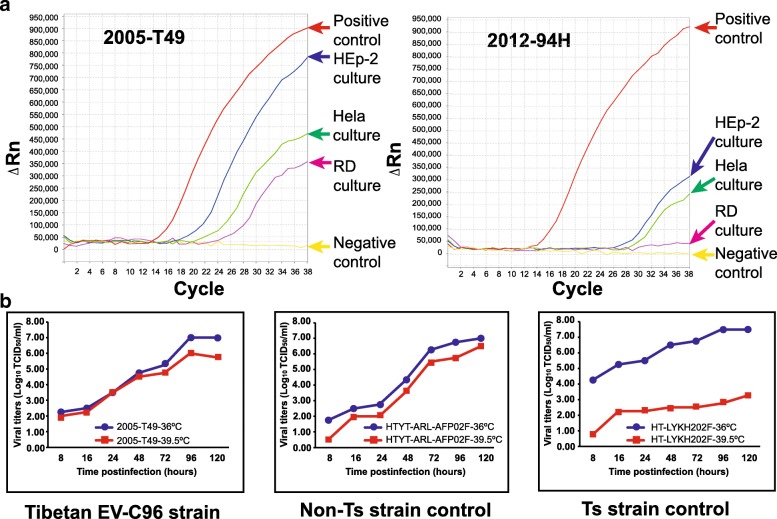
Real-time RT-PCR curve and temperature sensitivity test curve of two Tibetan EV-C96 strains. **a** Enterovirus RNAs in RD, Hep-2 and Hela cell cultures were detected with a real time RT-PCR-based enterovirus screening method. A Xinjiang EV-B85 strains, HYTY-ARL-AFP02F, was used as positive control, the RD cell culture without inoculating samples was used as negtive control. **b**. Strain 2005-T49 is a non-temperature sensitive (Non-*Ts*) strain. Two Xinjiang EV-B85 strains, HYTY-ARL-AFP02F, which is not temperature sensitive, and HT-LYKH202F, which is temperature sensitive (*Ts*), were used as controls. The blue and red lines show the growth curves of the viruses in RD cells at 36 °C and 39.5 °C, respectively

Tibetan EV-C96 strain 2005-T49 grew and showed clear CPE in RD cells, HEp-2 cells and Hela cells. In contrast, strain 2012-94H showed no CPE in these three cells. The results were similar when cultured at 33 °C and 36 °C. However, viral RNA of 2012-94H in HEp-2 and Hela cell culture were detected by real-time RT-PCR assay with the cycle threshold (Ct) values of 25.85 and 28.15, respectively, and RD cell culture was real-time RT-PCR negative, suggesting low replication compared with 2005-T49 (Ct values of 17.25, 20.05, and 22.20, respectively, in HEp-2, Hela, and RD cell culture) and indicating a different growth ability in cells for strain 2012-94H (Fig. 1a).

### The Tibet EV-C96 strains were not temperature sensitive

The replication capacity of Tibetan EV-C96 strain 2005-T49 at an elevated temperature (39.5 °C) was compared with that of two Xinjiang EV-B85 strains, HYTY-ARL-AFP02F, which is not temperature sensitive, and HT-LYKH202F, which is temperature sensitive [22]. The results showed that Tibetan EV-C96 strain 2005-T49 was not temperature sensitive, based on the results that a titre reduction of less than 2 logarithms at 36 °C/39.5 °C (Fig. 1b).

### Characterisation of the full-length genome sequence of the Tibetan EV-C96 strains

The full-length genome sequences of two Tibetan EV-C96 strains, 2005-T49 and 2012-94H, were obtained. The lengths of the 2005-T49 and 2012-94H genomes were 7471 and 7453 nucleotides, respectively, and both encoded a 2219-amino acid polypeptide. The coding sequences were flanked by non-coding 5′-UTRs of 744 and 726 nucleotides, respectively, and non-coding 3′-UTR of 70 nucleotides each followed by a poly (A) tail composed of a long sequence of adenine nucleotides. The 5′ end sequences obtained from the stool specimens are identical to those obtained from the viral isolates. Alignment of the two Tibetan EV-C96 full-length genomes with the genome of the EV-C96 prototype strain BAN00–10488 showed that they shared the same genomic organisation and same order of genomic regions. However, in the 5′-UTR, one of the Tibet EV-C96 strains, 2005-T94, contained two nucleotide deletions at positions 120 and 139, and strain 2012-94H contained an 18-nucleotide deletion at positions 713–730, which is located in complex RNA elements termed internal ribosome entry sites (IRESs) [30, 31]. In the *3A* region, both strains contained a 3-nucleotide deletion at position 5290–5292, and a 2-nucleotide deletion at 7407–7408 in the 3′-UTR. The overall base composition of strain 2005-T49 was 29.82% A, 22.94% G, 22.59% C, and 24.64% U, and that of 2012-94H was 29.99% A, 22.81% G, 22.55% C, and 24.65% U. The polypeptide cleavage sites were predicted based on the sequence of the EV-C96 prototype strain. The nucleotide and amino acid sequence identities in the *VP1*-coding region between strain 2005-T49 and 2012-94H were 87.3 and 94.4%, respectively, and 2005-T49 and 2012-94H respectively displayed 79.1 and 78.5% nucleotide identity and 93.5 and 92.5% amino acid identity in the *VP1* region with the prototype EV-C96 strain.

### Phylogenetic analysis of the Tibetan EV-C96 strains and other EV-C genomes

**Fig. 2:**
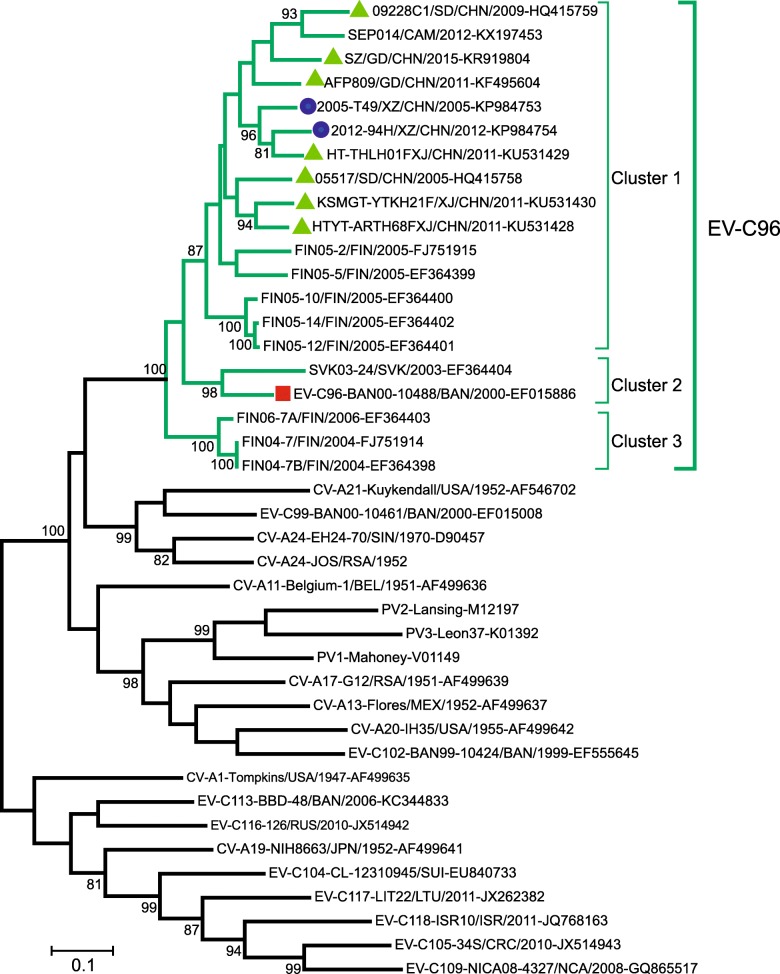
Molecular phylogenetic tree based on the *VP1* region of the EV-C strains. The evolutionary history of EV-C strains was inferred by using the maximum-likelihood method based on the Kimura 2-parameter model in MEGA7. The percentage of trees in which the associated taxa clustered together is shown next to the branches. EV-C96 prototype stain is indicated by a red square, the Tibetan EV-C96 strains are indicated by blue circles, and the other Chinese EV-C96 strains are indicated by green triangles

To investigate the phylogenetic relationships among the two Tibetan EV-C96 strains and other EV-C96 strains, a phylogenetic tree was generated based on the *VP1* region of the two Tibetan EV-C96 strains, all EV-C96 strains in GenBank, and all EV-C prototype strains (Fig. 2). In this tree, all EV-C96 strains were grouped with the prototype strain, and could be grouped into three clusters, as described previously [32]. One cluster (Cluster 1), contained strains from China, including all Chinese EV-C96 strains, Cambodia, and Finland; the second (Cluster 2), contained strains from Slovenia and Bangladesh as well as the prototype strain; and the third (Cluster 3) contained strains from Finland.

**Fig. 3:**
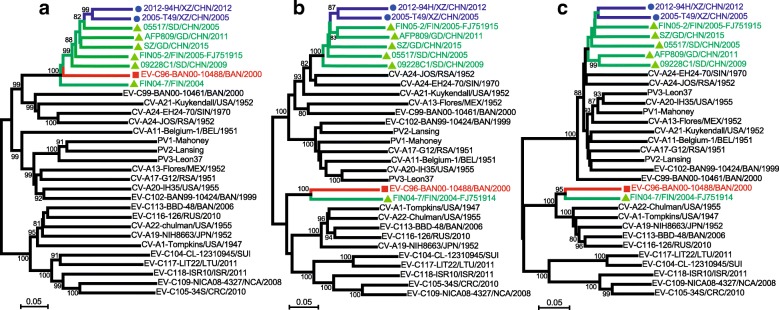
Phylogenetic relationships among enterovirus C (EV-C) strains based on the *P1*, *P2*, and *P3* regions. The Tibet EV-C96 strains described in this study (blue circles), other Chinese EV-C96 strains (green triangles), the EV-C96 prototype strain (red square), and 22 other EV-C prototype strains were analysed by nucleotide sequence alignment using the neighbour-joining algorithms in MEGA 7.0.26. The percentage of 1000 replicates in which the associated taxa clustered together in the bootstrap test is shown next to the branches. The scale bars represent the genetic distance. All panels have the same scale. **a** *P1* coding sequences, (**b**) *P2* coding sequences, and (**c**) *P3* coding sequences

Phylogenetic trees based on the *P1*, *P2*, and *P3* regions were also generated (Fig. 3). In the *P1* region-based tree, all EV-C96 strains were clustered together with the EV-C96 prototype strain. The serotype with the highest sequence similarity to the EV-C96 strains is CV-A24. No sub-cluster was observed because full-length genome sequences were not available for all EV-C96 strains. In the *P2* and *P3* region-based trees, the EV-C96 prototype strain, BAN-10488, and strain FIN04–7 formed a monophyletic cluster that did not group with any other EV-C96 strains or other EV-C types, and all Chinese EV-C96 strains were still clustered with CV-A24 and CV-A24v (Fig. 3). In all the trees mentioned above, the two Tibetan EV-C96 strains were close to each other, and were together with all other Chinese strains.

### Recombinant analysis of the EV-C96 strains

The phylogenetic analyses based on the *P2* and *P3* regions showed that the prototype EV-C96 strain BAN-10488 and the Chinese EV-C96 strains were on separate branches in a cluster formed by the EV-C prototypes. Strain BAN-10488 and the Chinese EV-C96 strains diverged by 28.65% in the *P2* and *P3* regions, and they diverged by 19.15–29.33% and 19.25–31.69%, respectively, from other EV-C prototype strains. Some recombination events must have occurred during EV-C96 evolution as EV-C96 isolates cluster with different EV-C prototype strains in phylogenetic trees in different genomic regions. However, recombination does not seem to have occurred frequently as EV-C96 isolates from different years and locations appear to cluster together in all genomic regions analysed.

A similarity analysis was also performed to investigate potential recombination in EV-C96 strains, and strain 2005-T49 were used as query sequences for comparison to the EV-C prototype strains. The similarity analysis demonstrated that the two Tibetan EV-C96 strains showed the highest sequence similarity with the EV-C96 prototype only in the *P1* region. However, in the *P2* and *P3* regions, the similarity between BAN-10488 and the Chinese EV-C96 strains was of the same magnitude as that between heterotypic EV-C prototype strains.

## Discussion

EV-C species viruses have been shown to be correlated with many diseases, such as poliomyelitis caused by poliovirus [33] and acute haemorrhagic conjunctivitis caused by CV-A24v [34]. In a previous study, EV-C-type viruses were clustered into three subgroups based on the *VP1*-coding region [35], and EV-C96 is clustered with CV-A21, CV-A24, EV-C95, and EV-C99. Notably, more than twenty cases of EV-C96 infection were reported in several provinces of China, including Shandong [17], Guangdong [32], Xinjiang [3], and Yunnan province [36], and most of these strains were isolated from patients with AFP, like in other countries, which suggests that EV-C96 may be an important etiological cause of AFP.

It was previously shown that different EV-C96 strains have different cell tropisms in RD and HEp-2 cells [32]. In this study, the two EV-C96 strains also showed different growth abilities in cells that differed from those of previously described EV-C96 strains. Although strain 2012-94H showed no CPE in RD, HEp-2 or Hela cells, however, viral RNA can be detected in HEp-2 and Hela cell cultures by real-time RT-PCR assay. Clearly, the virus could still replicate in the HEp-2 and Hela cells, it just did not produce CPE. So indeed, viral culture in immortalised cell lines does not necessarily have relevance to tissue tropism in vivo. The growth ability in cells could be affected by many things, changes in cell lines during repeated passages may have influenced the ability of 2012-94H to replicate in these cell lines. Therefore, further studies are needed to clarify the different growth abilities in different cells among different EV-C96 strains.

Interestingly, the full-length genome sequencing showed that strain 2012-94H has a deletion of 18 nucleotides in the IRES element in 5′-UTR. It is known that two major types of IRES element have been identified within picornaviruses, enteroviruses (include polioviruses and rhinoviruses) contain type I IRES, whereas the cardioviruses and foot-and-mouth disease viruses share another type of IRES, type II IRES. Although sequence identity between different members within type I IRES can be less than 50%, the secondary structure predictions are very similar [30, 37]. It has been shown that the 5′-UTR of enteroviruses plays an important role in RNA replication and the translation of viral proteins because of its cloverleaf-like secondary structure and IRES [38, 39]. Although there is not enough evidence yet, we hypothesise that this 18-nucleotide deletion in the 5′-UTR may affect viral replication, and may be one of the reasons why strain 2012-94H does not cause CPE in RD, HEp-2 and Hela cells. Our research team is currently using reverse genetic methods to elucidate the inherent mechanism of this interesting finding.

The full-length genome characterisation showed that the two Tibetan EV-C96 strains share high nucleotide sequence similarity with the EV-C96 prototype strain BAN00–10488 only in the *P1* region, which encodes the capsid protein of the virion. In contrast, in the non-structural coding regions *P2* and *P3*, the Tibetan strains showed higher genetic diversity when compared with EV-C prototype strains, including BAN00–10488, which is in agreement with the phylogenetic trees described above. EV-C96 strains from two different countries, China and Finland, showed a close phylogenetic relationship with each other, although there is no clear epidemiological evidence, this could be explained by the fact that the strains from Finland that belong to cluster 1 were thought to be imported from China [13]. The genetic diversity in the *VP1* region among the Chinese strains isolated in 2005–2012 indicated that EV-C96 has been circulating in China for many years and has been evolving.

Genetic recombination, an evolutionary pattern observed in most viruses, is a common phenomenon during the evolution of enterovirus [40, 41]. The junction between the *P1* region and the *P2* and *P3* regions may be a hotspot for recombination events, as exchanges here would not change the viral serotype or affect important functions [42]. The *P2* and *P3* regions of the Chinese EV-C96 strains were highly divergent from those of BAN-10488; in fact, the Chinese EV-C96 strains were divergent from all EV-C prototypes, and strain BAN-10488 was most similar to CV-A1/CV-A22/CV-A19. The difference between the Chinese EV-C96 strains and BAN-10488 was also evident in the phylogenetic trees based on the *P2* and *P3* regions, in which all Chinese EV-C96 strains formed a separate cluster, and the BAN-10488 clustered with CV-A1/CV-A22/CV-A19.

In this study, the full-length genomes of two EV-C96 strains isolated from the Tibet Autonomous Region of China were characterised, and their phylogenetic relationships with other EV-C96 strains and other EV-C serotypes were analysed. The different growth patterns on RD, HEp-2 and Hela cells among EV-C96 strains suggest a unique pattern of phenotypic characterisatics among viruses of this type, one of the reasons leading to this phenotypic character may be due to a deletion of 18 nucleotides in the IRES element in 5′-UTR, which is worthy of further study. There is genetic variability between EV-C96 strains which suggest that at least few genetic lineages co-exist and there has been some degree of circulation in different geographical regions for some time. The three clusters of EV-C96 in the *P1*-based tree may represent three different genotypes, and all Chinese EV-C96 strains belong to cluster 1. These findings expand the understanding of the characterization of EV-C96 and are meaningful for the surveillance of the virus.

## Abbreviations

AFP: Acute flaccid paralysis
CCID_50_: 50% cell culture infectious dose
CPE: Cytopathic effects
Ct: Cycle threshold
EV: Enterovirus
EV-C96: Enterovirus C96
Hela cell: Human cervical cancer cell
HEp-2 cell: Human laryngeal epidermoid carcinoma cell
IRES: Internal ribosome entry site
RD cell: Human rhabdomyosarcoma cell
UTR: Untranslated regions
